# Notes on Preparations for the Sick

**Published:** 1913-09

**Authors:** 


### Notes on Preparations for the Sick.

**Fortovim.**—HODDER & Co., Bristol.

**Visem.**—ST. IVEL LTD., Yeovil.

To the making of varieties of milk foods there seems to be no limit.

Under the name Fortovim we find a concentrated proteid food, which is stated to be free from starch, sugar and fat, but containing glycero-phosphates. Our examination showed only traces of fat, but the absence of this food constituent is not always an advantage. The main constituent is quite obviously milk proteid.

Visem is extensively advertised as a tonic milk food, containing lecithin, and our analysis confirms this statement. A small percentage of fat is usual in such preparations, but the bulk of this component of the original milk has been removed.

---

**Validol and Compounds.**—ZIMMER & Co., Frankfort-on-Main. —This isovalierianic-menthol ester has now been in use for some fifteen years, and has been found useful for a great variety of nerve conditions. For sea-sickness a dosage of 5 to 10 drops every hour, and as a nerve tonic 5 drops on sugar or in spirit may be taken three times a day.

It is mounted also in rose-coloured pearls, in tablets, chocolates, bon-bons, as an effervescent salt, with 10 per cent. of camphor as Validol-camphoratum, and as Brom-Validol tablets, containing 15 grains of sodium bromide.

---

**Helmitol Co. Tablets. Hydrastinin-hydrochlor Tablets.**—THE BAYER COMPANY, London.

Tablets "Helmitol" Co. contain equal parts of helmitol and acid sodium phosphate, and have been introduced at the express request of a number of medical men, in whose experience the addition of the acid phosphate is advisable in certain conditions associated with pronounced alkalinity of the urine. Especial importance has been attached to the use of the compound in bacillus coli cases.

The dose is two to three tablets dissolved in plenty of cold water three times a day, and the tablets are dispensed in original tubes of twenty.

Tablets Hydrastinine HCl.—This preparation is of great interest, as the synthesis of the complicated alkaloid is looked upon as a chemical achievement. The alkaloid has been found to be in every way identical with that obtained by oxidation from the hydrastine of the rhizome.

The tablets have the advantage over the ordinary galenical preparations of hydrastis of definite composition, while it is now recognised that hydrastinine has advantages over hydrastine, that its styptic action is more powerful and lasting, that it is less depressing to the heart, and that it does not cause tetanic spasms. The tablets, which are silver-coated and packed in tubes of fifteen, are of special service in checking hemorrhages and discharges. The dose is one tablet swallowed with a draught of water three or four times a day.

**Kalari Biscuits.**—Callard & Co., London.—These are palatable biscuits, to be eaten under the same condition as ordinary bread and for use instead of bread and toast. They are free from starch and sugar, consisting almost entirely of milk proteids and vegetable albuminoids. The relative value of bread and toast is shown by the following analysis :—

| | Wheaten Bread. | Toasted Bread. | Kalari Biscuits. |
|---|---|---|---|
| Starch, dextrine, etc. .. | 50.0 | 85.5 | .0 |
| Moisture .. .. .. .. .. | 40.0 | .0 | 8.1 |
| Fat .. .. .. .. .. .. .. | .8 | 1.2 | 26.6 |
| Cellulose .. .. .. .. .. | .7 | 1.0 | .0 |
| Albuminoids .. .. .. .. | 7.2 | 10.5 | 60.8 |
| Mineral matter .. .. .. | 1.3 | 1.8 | 4.5 |
| | 100.0 | 100.0 | 100.0 |

Bread loses moisture when toasted ; the proportion of the fat-producing constituents (carbo-hydrates) is thus increased. Toast is therefore more fattening than bread, unless it is burnt almost to a cinder ; it is then charcoal and unfit for food.

---

**Wander Malt Extract, with Iron Iodide. Wander Malt Extract, with Hæmoglobin.**—A. Wander Ltd., London, E.C.—These samples are crystalline powders having the composition indicated.

"Wander" Malt Extract (Crystalline) with Iron Iodide is an elegant and exceedingly palatable product. It does not oxidise, and therefore gives perfect security of correct dosage and rapid absorption of ferrous iodide. In addition to this, the associated malt extract prevents any symptoms of iodism or gastric disturbance, corrects astringency, and enhances the value of the treatment with its digestive and flesh-forming properties.

This preparation affords a means of administering that valuable remedy ferrous iodide in a perfectly stable and satisfactory manner, for the disadvantages of the official syrup and other preparations have been overcome. It may be advantageously prescribed wherever ferrous iodide is indicated, such as tuberculous conditions of the glands, bones and joints, various forms of anæmia, and in certain rheumatic affections.

Of the Hæmoglobin compound a full dose of one tablespoonful contains 3 gramms. (46½ grains) of pure Hæmoglobin.

Other combinations of this malt extract are prepared with guaiacol carbonate, with glycero-phosphates and with iron pyro-phosphate.

**Œthone-Falcoz.**—WILCOX, JOZEAU & CO., London.—This alcoholic liquid, belonging to the carberine series, has the composition $C_7H_{16}O_3$. It exerts a rapid and efficacious influence on all kinds of spasmodic cough, and therefore is of value in cases of whooping cough. Thirty to fifty drops taken in syrup and mixed with water just before its use is a valuable remedy in many varieties of cough due to chronic bronchitis, asthma and tuberculosis.

---

**Winox.**—WINOX LTD., 65 London Wall, E.C.—This wine-food is described as the best tonic wine, containing the natural elements of sound wine, English beef and pure malt. Its analysis is given as follows :—

| | |
|---|---|
| Specific gravity .. .. | 1062.20 |
| Alcohol (by vol.) .. .. | 19.12 per cent. |
| Solids .. .. .. .. .. | 20.42 ,, |
| Nitrogen .. .. .. .. | 0.111 ,, |
| Mineral constituents.. .. | 0.74 ,, |
| Phosphoric acid .. .. | 0.168 ,, |
| Equal to | |
| Phosphate of lime .. .. | 0.367 ,, |

"The Company claim that by concentrating the grape juice before preparing the wine from which 'Winox' is made its value could be greatly increased, and that it is proved by analysis that wine thus made contains three times the normal amount of natural phosphorus."

